# Collaborative Enhancement of Antibody Binding to Distinct PECAM-1 Epitopes Modulates Endothelial Targeting

**DOI:** 10.1371/journal.pone.0034958

**Published:** 2012-04-13

**Authors:** Ann-Marie Chacko, Madhura Nayak, Colin F. Greineder, Horace M. DeLisser, Vladimir R. Muzykantov

**Affiliations:** 1 Department of Radiology, Division of Nuclear Medicine and Clinical Molecular Imaging, Perelman School of Medicine, University of Pennsylvania, Philadelphia, Pennsylvania, United States of America; 2 Department of Emergency Medicine, Perelman School of Medicine, University of Pennsylvania, Philadelphia, Pennsylvania, United States of America; 3 Pulmonary, Allergy & Critical Care Division, Perelman School of Medicine, University of Pennsylvania, Philadelphia, Pennsylvania, United States of America; 4 Institute for Translational Medicine and Therapeutics, Perelman School of Medicine, University of Pennsylvania, Philadelphia, Pennsylvania, United States of America; Genentech, United States of America

## Abstract

Antibodies to platelet endothelial cell adhesion molecule-1 (PECAM-1) facilitate targeted drug delivery to endothelial cells by “vascular immunotargeting.” To define the targeting quantitatively, we investigated the endothelial binding of monoclonal antibodies (mAbs) to extracellular epitopes of PECAM-1. Surprisingly, we have found in human and mouse cell culture models that the endothelial binding of PECAM-directed mAbs and scFv therapeutic fusion protein is increased by co-administration of a paired mAb directed to an adjacent, yet distinct PECAM-1 epitope. This results in significant enhancement of functional activity of a PECAM-1-targeted scFv-thrombomodulin fusion protein generating therapeutic activated Protein C. The “collaborative enhancement” of mAb binding is affirmed *in vivo*, as manifested by enhanced pulmonary accumulation of intravenously administered radiolabeled PECAM-1 mAb when co-injected with an unlabeled paired mAb in mice. This is the first demonstration of a positive modulatory effect of endothelial binding and vascular immunotargeting provided by the simultaneous binding a paired mAb to adjacent distinct epitopes. The “collaborative enhancement” phenomenon provides a novel paradigm for optimizing the endothelial-targeted delivery of therapeutic agents.

## Introduction

Drug targeting to endothelial cells (ECs) (i.e., “vascular immunotargeting”) has the potential to improve management of diseases involving ischemia, inflammation, thrombosis, and tumor growth [1]–[5]. In particular, conjugation of therapeutics with antibodies to PECAM-1 (platelet endothelial cell adhesion molecule 1, CD31) enables their endothelial delivery, boosting specificity and efficacy of their action in animal models [3], [6]. Further optimization of this promising approach is warranted to support translation into the clinical domain.

PECAM-1, a 130-kDa glycoprotein with six extracellular Ig-like domains, a transmembrane domain and a cytoplasmic tail (**Figure S1**), is present at modest levels on platelets and leukocytes [7], and is highly expressed on ECs (10^6^ copies per cell) [7], [8]. Endothelial PECAM-1 molecules engage in *trans* (i.e., antiparallel) homophilic interactions at intercellular junctions *via* distal Ig-like domain 1 (IgD1) and domain 2 (IgD2) [9], [10], and are involved in maintenance of EC monolayer integrity [11], mechanosensing [12], and cellular signaling [13]. Endothelial PECAM-1 also facilitates leukocyte migration *via* homophilic and heterophilic interactions with leukocytic PECAM-1 and other binding ligands [14].

Monoclonal antibodies (mAbs) directed to different extracellular epitopes and domains of PECAM-1 have been used as probes to study the role of PECAM-1 in mediating homophilic and heterophilic binding interactions [9], [10], [15]–[18], as well as affinity ligands for endothelial targeting of drugs, and nanocarriers [3], [19]–[21]. Antibodies directed to distinct PECAM-1 epitopes have different functional effects, either inhibiting, augmenting, or having no effect on the IgD1/IgD2-mediated homophilic binding interactions of PECAM-1 [17], [22]. Further, the engagement of specific PECAM-1 epitopes controls the rate of endothelial internalization and intracellular trafficking of nanocarriers targeted by PECAM-1 mAbs [23]. These results suggest that optimization of immunotargeting and intracellular delivery is possible through the engagement of distinct PECAM-1 epitopes.

In the present study we set out to investigate the *in vitro* and *in vivo* binding parameters of mAbs directed to the IgD1 and IgD2 domains of PECAM-1 and address mutual effects of their binding. The latter aspect is a relatively uncharted one in vascular immunotargeting. Studies in this area are limited to mAbs to angiotensin-converting enzyme (ACE), a promising molecular target for drug delivery to endothelium [24], [25], and show that anti-ACE mAbs directed to distinct epitopes negatively mutually interfere with binding of each other [26].

However, in contrast with this somewhat expected outcome with anti-ACE mAbs, our results indicate that endothelial immunotargeting of anti-PECAM-1 mAb can be significantly enhanced by the simultaneous binding of paired mAbs directed to adjacent, yet distinct PECAM-1 epitopes in both *in vitro* cell culture and *in vivo* mouse studies. Motivated by this hugely unusual outcome, we set out to determine whether augmentation in binding translates to an increase in therapeutic protein delivery and functional output. We used a therapeutic fusion protein targeted to PECAM-1 to demonstrate that enhanced delivery results in a significant increase in the fusion-catalyzed generation of a cell-protective species with antithrombotic and anti-inflammatory activities. This antibody-dependent “collaborative enhancement” phenomenon illustrates the potential of this targeting strategy for increasing the efficiency of vascular delivery in therapeutic applications.

## Results

### Characterization of in vitro PECAM-1 interactions with mAbs

**Figure 1:**
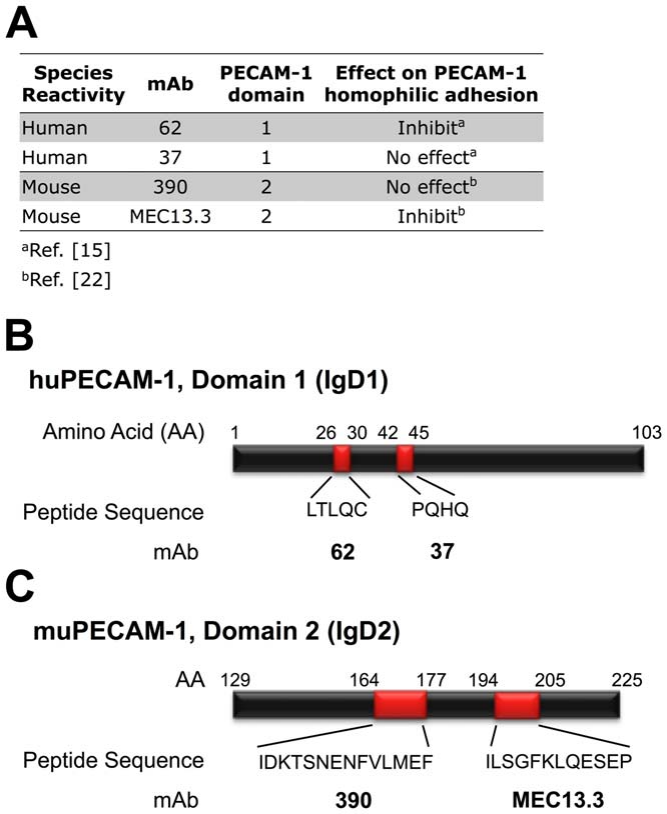
Monoclonal antibody (mAb) ligands recognizing distinct extracellular epitopes of PECAM-1. (**A**) MAbs investigated in this study to probe the affinity and accessibility to distinct epitopes of human PECAM-1 (huPECAM-1; mAbs 62 and 37) and mouse PECAM-1 (muPECAM-1; mAbs 390 and MEC13.3). Listed is the effect of various anti-PECAM-1 mAbs on PECAM-1-dependent homophilic adhesion, as defined by the aggregation of L-cells fibroblast transfectants expressing PECAM-1 [22], [50]. [15], [22]. (**B**–**C**) Diagram of immunoreactive regions within PECAM-1 domains 1 and 2. (**B**) Amino acid (AA) location of distinct non-overlapping epitopes for binding of mAbs 62 and 37 on Ig-domain 1 (IgD1) of huPECAM-1 [22]. (**C**) AA location of epitopes for mAbs 390 and MEC13.3 on Ig-domain 2 (IgD2) of muPECAM-1 (H. DeLisser, unpublished results). Peptide sequence recognized by mAbs are colored in red.

**Figure 2:**
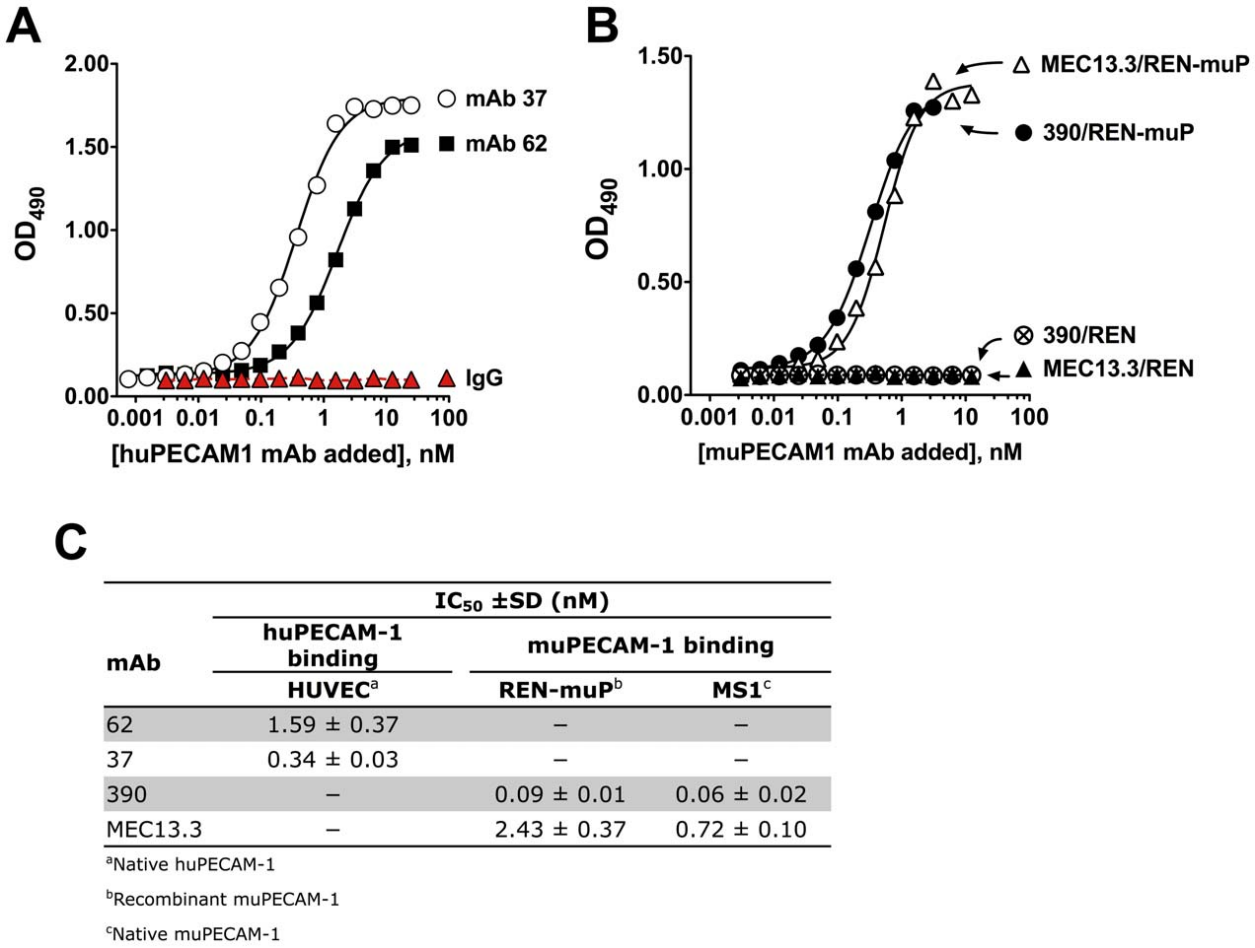
*In vitro* binding properties of mAb to live cells expressing PECAM-1. Cell surface binding of mAbs to PECAM-1 was determined by ELISA-based method with (**A**) HUVECs, (**B**) REN-muP cells. Proteins were added to confluent cellular monolayers at the indicated dilutions and incubated for 2 h at 4°C. The results shown are from a representative experiment. Non-targeted IgG or non-PECAM-1 expressing cells were used as negative control. Representative plots for mAb binding to MS1 cells are available in **Figure S2**. (**C**) Analysis of the relative binding affinity of anti-PECAM-1 mAbs, when binding to cells is half-maximal (IC_50_). Data points were fit as described under “Methods.” The IC_50_ is reported as the mean IC_50_ value ± SD of three independent experiments performed in triplicate.

Epitope mapping has shown that mAbs 62 and 37 bind to distinct epitopes in IgD1 in human PECAM-1 (huPECAM-1) [22], and mAbs 390 and MEC13.3 bind to their respective non-overlapping epitopes in IgD2 of the murine homolog, muPECAM-1 (H. DeLisser, unpublished results; **Figure 1**). The specificity and sensitivity of these mAbs for binding to PECAM-1 was confirmed by live-cell ELISA using confluent monolayers of human endothelial cells (human umbilical vein endothelial cells (HUVECs)) and human endothelial-like REN cells stably expressing recombinant muPECAM-1 (REN-muP) [27]. In these cell culture models, most of surface PECAM-1 molecules are involved in *trans-*homodimeric interactions at intercellular borders [28]–[30]. ELISA showed that unmodified anti-PECAM mAbs specifically bind to ECs at nanomolar levels, albeit with considerable differences in binding, as reflected by IC_50_ (**Figure 2**). In HUVECs, mAb 62 binding is ∼5−fold weaker vs mAb 37 binding (IC_50_ = 1.59 nM vs 0.34 nM) (**Figure 2C**). Further, mAb MEC13.3 binding to REN-muP cells (IC_50_ = 2.43 nM) is 27−fold weaker than the mAb 390 binding (IC_50_ = 0.09 nM) (**Figure 2C**). The binding of mAb MEC13.3 is 12−fold lower than mAb 390 to MS1 cells expressing native muPECAM-1 (**Figure 2C**, **Figure S2**).

**Figure 3:**
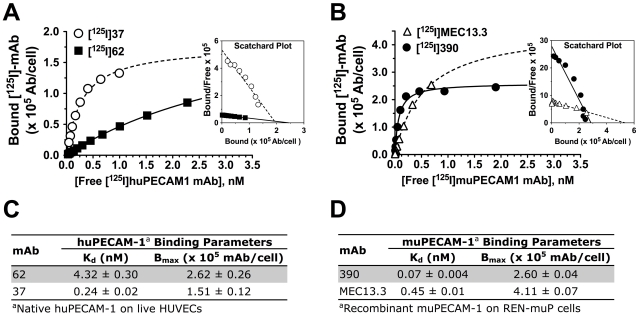
Binding parameters of anti-PECAM-1 [^125^I]-mAbs to live cells expressing PECAM-1. Cell surface binding parameters (K_d_ and B_max_) of [^125^I]-mAbs to PECAM-1 was determined by RIA-based method with (**A**) native huPECAM-1 on HUVECs, and (**B**) recombinant muPECAM-1 on REN-muP cells. Serial dilutions of [^125^I]-mAbs were added to confluent cellular monolayers and incubated for 2 h at 4°C. The results shown are from a representative experiment, with the inset showing Scatchard plot of binding data. Note that total binding was corrected for NSB using 100−fold excess of unlabeled mAb for HUVECs or using parent REN cells for REN-muP binding. (**C**–**D**) K_d_ and B_max_ Binding parameters are for [^125^I]-mAbs to huPECAM-1 and muPECAM-1 are listed. Results were determined by three independent RIA experiments performed in quadruplicate, with data expressed as mean ± SD.

Live-cell radioimmunoassay (RIA) of ^125^I-labeled mAbs ([^125^I]-mAb) was used for quantitative assessment of equilibrium binding parameters (K_d_), including the number of maximum available binding sites (B_max_). Analysis of [^125^I]-mAb binding to HUVECs by RIA yielded K_d_ of 4.32 nM and 0.24 nM for [^125^I]-mAb 62 and [^125^I]-mAb 37, respectively (corresponding B_max_ values are 2.6×10^5^ mAb/cell and 1.5×10^5^ mAb/cell) (**Figure 3A**). [^125^I]-MAb 390 and [^125^I]-mAb MEC13.3 specifically bind to REN-muP cells with K_d_ 0.07 nM and 0.45 nM, respectively (corresponding B_max_ values are 2.6×10^5^ mAb/cell and 4.1×10^5^ mAb/cell) (**Figure 3B**). Similarly, [^125^I]-mAb 390 and [^125^I]-mAb MEC13.3 specifically bind to MS1 ECs with K_d_ 0.25 nM and 2.81 nM, respectively, and with B_max_ of mAb 390 also being nearly twice lower that mAb MEC13.3 (**Table S1**).

### Modulation of in vitro PECAM-1 targeting

**Figure 4:**
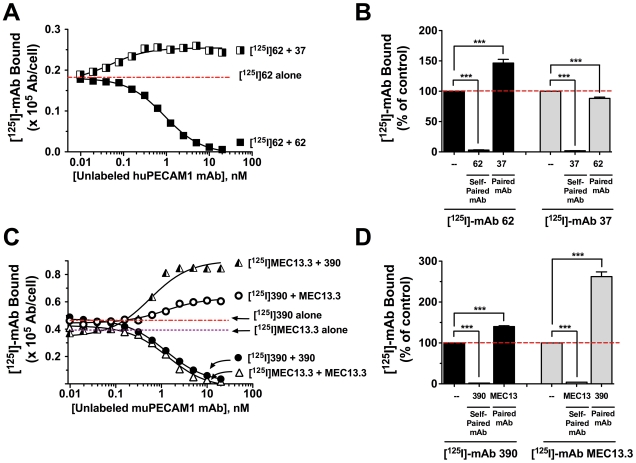
Anti-PECAM-1 [^125^I]-mAb binding in live cells is enhanced by paired mAb directed to adjacent PECAM-1 epitope. The modulation of PECAM-1 binding was determined by co-incubation of [^125^I]-mAb with indicated concentrations of unlabeled self-paired mAb or paired mAb with cells for 2 h at 4°C. Binding data were plotted as [^125^I]-mAb molecules bound per cell (mAb/cell) and data points were fit as described under “Methods.” (**A** and **B**) Unlabeled mAb 62 competitively inhibits binding of [^125^I]-mAb 62 to huPECAM-1 in HUVEC. However, mAb 37 enhances [^125^I]-mAb 62 binding to huPECAM-1 in HUVEC by 1.5−fold over binding of [^125^I]-mAb 62 alone. Interestingly, mAb 62 does not enhance the binding of [^125^I]-mAb 37 (**Figure S3**). (**C**–**D**) Collaborative binding studies of mAbs 390 and MEC13.3 with REN-muP cells as described in panel A. Unlabeled self-paired mAb 390 and mAb MEC13.3 competitively inhibit binding of [^125^I]-mAb390 and [^125^I]-mAb MEC13.3 to REN-muP cells, respectively. In contrast, mAb pairs [^125^I]-mAb 390/MEC13.3 and [^125^I]-mAb MEC13.3/390 enhance binding by ∼1.5−fold and ∼2.7−fold, respectively, over [^125^I]-mAb alone (***, P<0.001, *n* = 3–4).

We next investigated the mutual binding effects of mAb 37 and 62 to their epitopes in IgD1 of huPECAM-1. Expectedly, endothelial binding of [^125^I]-mAb 62 and [^125^I]-mAb 37 was competitively inhibited by their respective unlabeled mAb counterparts directed to the same epitope (“self-paired”) (**Figure 4A**; **Figure S3**). However, binding of [^125^I]-mAb 62 was enhanced 1.5−fold by unlabeled mAb 37 (“paired”) (**Figure 4A,B**). This enhancement effect was not mutual, as unlabeled mAb 62 did not alter the binding of [^125^I]-mAb 37 (**Figure S3**). [^125^I]-mAbs 62 and 37 bind to immobilized huPECAM-1, but not to mAb pairs or control IgG (**Figure S4**). This result confirms that modulation of anti-PECAM mAb binding to endothelial cells is due to binding through cellular PECAM-1 and not due to binding to cell-associated antibodies.

RIA of [^125^I]-mAb 62 co-incubated with 50 nM enhancer mAb 37 with HUVEC revealed that the apparent binding affinity of [^125^I]-mAb62 is increased nearly 1.4−fold (K_d_ 4.25 nM→2.96 nM, P<0.001) (**Table S2**). Furthermore, a similar result is observed using wells coated with the soluble extracellular domain of recombinant huPECAM-1: the apparent binding affinity of [^125^I]-mAb62 with mAb 37 co-treatment increases nearly four−fold (K_d_ 4.77 nM→1.24 nM, P<0.001) (**Table S2**). Taken together, these data suggest that the modulation of [^125^I]-mAb62 binding by an enhancer mAb, as evidenced by changes in K_d_ and B_max_, is mediated specifically through huPECAM-1 *via* collaborative enhancement. It remains unclear at this time if the observed phenomenon is due to changes in a single PECAM-1 molecule or changes in homodimeric PECAM-1-PECAM-1 interactions.

To test whether the collaborative binding phenomenon is unique to human PECAM-1, we investigated mAb modulatory effects on muPECAM-1-expressing cells. Binding of [^125^I]-mAb 390 and [^125^I]-mAb MEC13.3 to REN-muP cells expressing recombinant muPECAM-1 was inhibited by its unlabeled self-paired mAb, yet enhanced by paired mAb directed to a distinct muPECAM-1 epitope (**Figure 4C**). These results were recapitulated in murine MS1 endothelial cells expressing native muPECAM-1 (**Figure S5**). Interestingly, the most dramatic collaborative enhancement was observed with the pairing of mAb 390 (K_d_ = 0.07 nM) with [^125^I]-mAb MEC13.3 (K_d_ = 0.45 nM), resulting in a 2.7−fold increase in binding over [^125^I]-mAb MEC13.3 alone (**Figure 4C,D**). MAb MEC13.3, with its 6−fold lower affinity relative to mAb 390 was able to enhance [^125^I]-mAb 390 binding up to 1.5−fold above control uptake.

### Collaborative enhancement increases targeting and effect of a therapeutic fusion protein

**Figure 5:**
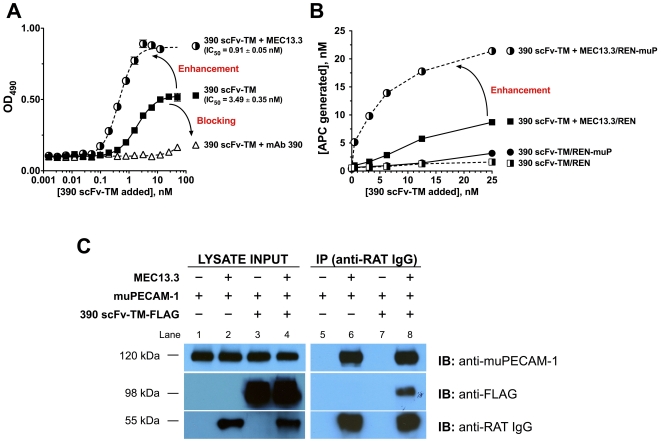
In vitro enhancement of binding, accessibility and therapeutic output of anti-PECAM-1 390 scFv-TM fusion protein *via* dual epitope-engagement of muPECAM-1. (**A**) Cell surface binding of the therapeutic fusion protein 390 scFv-TM to REN-muP cells was assessed in the presence of 200 nM self-paired parental mAb 390 or paired mAb MEC13.3 by ELISA. The curves shown are representative ELISA. Only binding to REN-muP cells shown; there was no significant binding detected using control REN cells lacking muPECAM-1. Binding affinity of 390 scFv-TM, reflected by IC_50_, increases 3.8−fold when paired with MEC13.3. The IC_50_ is reported as the mean IC_50_ value ± SD of three independent experiments performed in triplicate. (**B**) Generation of activated protein c (APC), a cell-protective species, on the surface of REN-muP cells is initiated by targeted binding of 390 scFv-TM (+thrombin). APC generation is augmented up to 5−fold when 390 scFv-TM binding is enhanced with paired mAb MEC13.3 compared to 390 scFv-TM alone. (**C**) Co-IP of the MEC13.3/muPECAM-1/390 scFv-TM-FLAG complex in REN-muP cells. REN-muP cells were treated with muPECAM-1 targeted rat anti-mouse IgG MEC13.3 and anti-mouse 390 scFv-TM-FLAG combinations. Cell lysates were immunoprecipitated with Protein G agarose beads to MEC13.3 and analyzed by SDS-PAGE and immunoblotting (IB) using anti-muPECAM-1, anti-FLAG, and rat polyclonal anti-mouse antibodies, as described under “Methods.” For controls, REN-muP cells ±390 scFv-TM FLAG were incubated with Protein G beads alone (lanes 1 and 5, 3 and 7). 390 scFv-TM-FLAG was only detected in the IP for REN-muP cells co-treated with MEC13.3 and 390 scFv-TM-FLAG (lane 6), indicating an interaction between MEC13.3 and 390 scFv-TM through muPECAM-1. Data are representative of two independent experiments.

Collaborative enhancement of anti-PECAM mAbs binding was validated using a novel protein therapeutic prodrug, i.e., the extracellular domain of mouse thrombomodulin (TM) fused to a single-chain variable fragment (scFv) targeted to the 390 epitope of muPECAM-1 (390 scFv-TM [31]). Live-cell ELISA demonstrated that paired mAb MEC13.3 increased the apparent binding affinity of 390 scFv-TM ∼4−fold relative to fusion alone (IC_50_ 0.91 nM vs. 3.49 nM) (**Figure 5A**). Self-pairing the epitope with maternal mAb 390 inhibited 390 scFv-TM binding close to control levels with REN cells. This increase in binding affinity is accompanied by an increase in 390 scFv-TM bound to muPECAM-1, as made apparent by a higher maximum OD_490_ value compared to 390 scFv-TM alone.

We further examined whether enhanced delivery of 390 scFv-TM may have therapeutic consequences. TM captures the serine-protease thrombin and modulates its pro-thrombotic activity to convert protein C to activated protein C (APC), which itself has cell-protective anti-thrombotic and anti-inflammatory effects [32]. Targeting of the TM fusion protein to the luminal endothelial surface helps to control coagulation and inflammation in animal models of acute lung injury and ischemia/reperfusion *via* APC-mediated pathways [3], [31]. 390 scFv-TM bound to REN-muP cells, which have no endogenous TM, generates APC from protein C zymogen in the presence of thrombin. We found that REN-muP cells co-incubated with 390 scFv-TM and MEC13.3 demonstrated a ∼6−fold increase in APC generation relative to 390 scFv-TM alone (**Figure 5B**). Moreover, pairing of mAb MEC13.3 with 390 scFv-TM seemed to shift the potency of the prodrug (based on APC generation levels) to lower concentrations of 390 scFv-TM. These observations closely parallel the ELISA results and indicates an increase in both binding affinity and absolute fusion protein bound.

Co-immunoprecipitation (co-IP) studies revealed formation of a tri-molecular complex between 390 scFv-TM, PECAM-1 and MEC13.3 mAb (**Figure 5C** **, lane 8**). The simultaneous binding of the antibody ligands to adjacent non-overlapping epitopes of PECAM-1 suggests that the increased binding and functional effect of the fusion protein are mediated through modulation of its interaction with PECAM-1 by the enhancing antibody.

### In vivo PECAM-1 targeting

**Figure 6:**
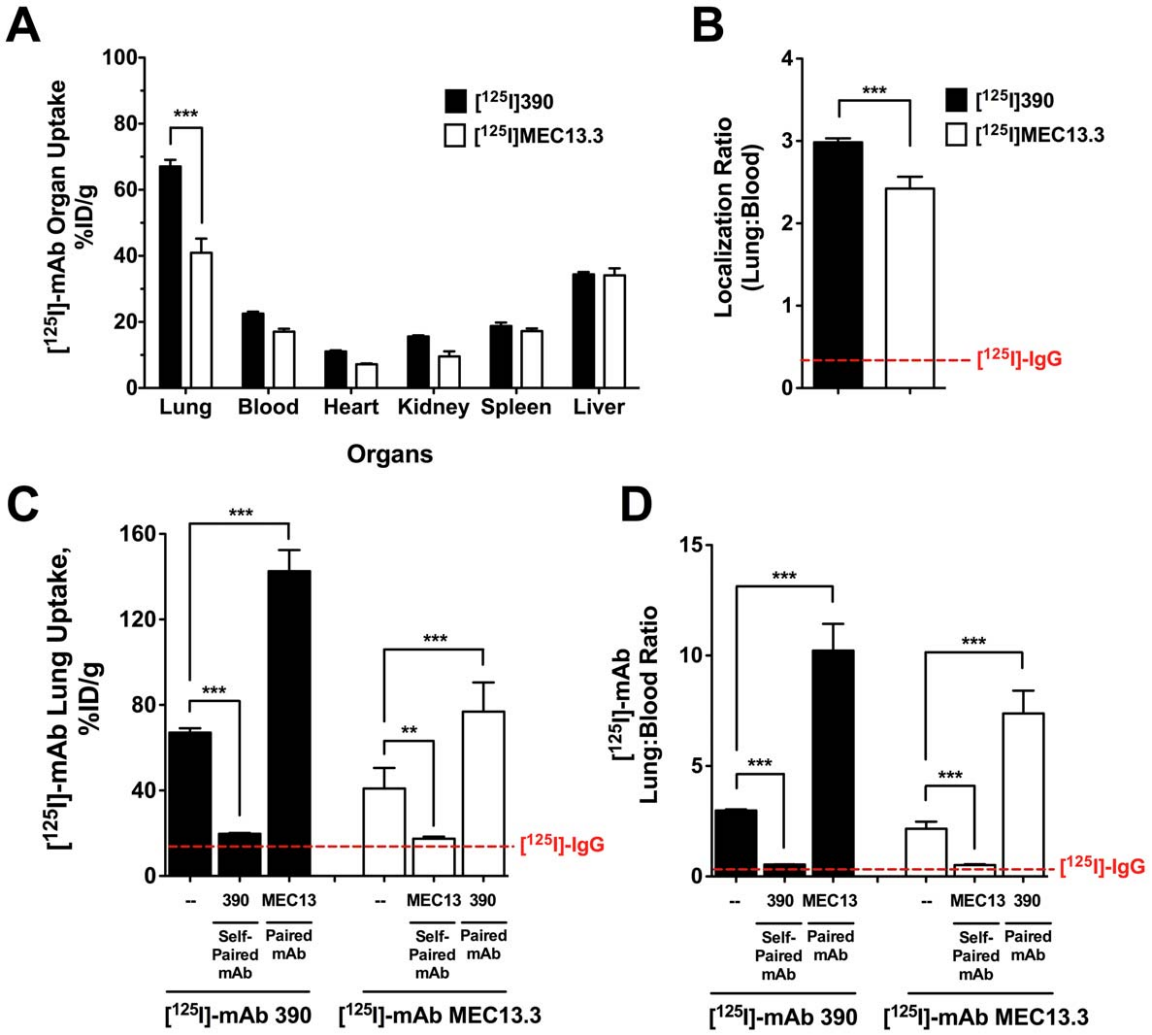
*In vivo* endothelial targeting of [^125^I]-mAb to muPECAM-1 is enhanced by paired muPECAM-1 mAb. (**A**) Biodistribution of anti-muPECAM-1 [^125^I]-mAbs 390 and MEC13.3 (0.2 µg/mouse) 30 min post-injection. (**B**) Localization ratio (LR) of [^125^I]-mAb pulmonary uptake normalized to residual blood radioactivity (lung∶blood), reflecting selectivity of PECAM-1-directed targeting to vascular endothelium. (**C**) Pulmonary uptake of [^125^I]-mAb 390 and [^125^I]-mAb MEC13.3 in inhibited by co-injection of unlabeled self-paired mAb (30 µg/mouse) directed to the same epitope. Co-injection of a paired mAb (30 µg/mouse) enhanced targeting of both [^125^I]-mAb 390 and [^125^I]-mAb MEC13.3 by 2.1− and 1.9−fold, respectively. The dashed red line indicates the level of non-specific [^125^I]-IgG uptake in the lungs. (**D**) Lung∶blood LR for [^125^I]-mAb 390/mAb MEC13.3 and [^125^I]-mAb MEC13.3/mAb 390 pairs increases 3.4−fold. The dotted red line is the LR of [^125^I]-IgG at 30 min p.i. Data is reported as the mean ± SEM of *n* = 4–5 animals (***, P<0.001; **, P<0.01).

*In vitro* studies suggest that mAb-mediated modulation of endothelial binding may have important implications for the vascular immunotargeting using PECAM-1 antibodies. To evaluate collaborative enhancement of immunotargeting *in vivo* and recapitulate cell culture findings, we studied effects of non-labeled mAbs on the pulmonary uptake of [^125^I]-mAb 390 and [^125^I]-mAb MEC13.3 injected in mice (**Figure 6**). The pulmonary vasculature, due to the privileged perfusion and extended endothelial surface area [33], is the preferential target of mAbs directed to PECAM-1 [3], [6]. Pulmonary targeting of [^125^I]-mAb 390 and [^125^I]-mAb MEC13.3 alone was reconfirmed and determined to be 67% ID/g and 41% ID/g, respectively (**Figure 6A,B**). Subsequently, [^125^I]-mAbs were co-administered with self-paired or paired mAb, and the *in vivo* results recapitulated cell culture findings. The pulmonary uptake of [^125^I]-mAbs was inhibited by co-injection of non-labeled self-paired mAb down to levels observed with control [^125^I]-IgG. Co-administration of paired mAb led to 2.1−fold and 1.9−fold enhancement in the pulmonary uptake of [^125^I]-mAb 390 and [^125^I]-mAb MEC13.3, respectively (**Figure 6C**). Correcting pulmonary uptake levels for residual blood activity yields a more accurate reflection of collaborative enhancement due to active vascular immunotargeting of anti-PECAM-1 mAb. As compared to [^125^I]-mAbs alone (**Figure 6B**), the lung∶blood localization ratio for both muPECAM-1 mAb pairs is enhanced 3.4−fold over mAb alone (**Figure 6D**).

## Discussion

The binding of ligands, including antibodies to epitopes of target molecules can block the delivery of ligands directed to the same epitope, or potentially modulate (i.e., block or enhance) the binding of ligands directed to secondary epitopes. Herein, we examined the interaction of a panel of four monoclonal antibodies (mAbs) directed to distinct extracellular epitopes of PECAM-1 domains IgD1 (human) and IgD2 (murine) (**Figure 1**) for understanding and optimizing endothelial immunotargeting. PECAM-1 mAb binding exhibits properties characteristic of mAb-antigen interactions: high affinity and specificity contributed by the steric complementarity between the antibody and antigen surface (**Figures 2** **,** **3**). Interestingly, for the mAbs evaluated it was clear that not all epitopes are displayed on PECAM-1 equally. In this study, we found that the mAb with higher affinity was accompanied by lower epitope accessibility, as reflected by B_max_ (**Figure 3B**). Variable accessibility to different antibodies could result from differences in: (1) masking of an epitope (e.g., due to tertiary structure of Ig-like domain, or masking by protein glycosylation and/or other components of the plasmalemma), (2) protein associations (e.g., different cell surface distribution and/or cytoskeletal associations), (3) membrane turnover of PECAM-1 sub-populations, or (4) Ab-induced shedding of PECAM-1 resulting in diminished epitope expression. However, K_d_ and B_max_ binding parameters can serve as valuable empiric criteria in judiciously selecting the most effective ligand (i.e. high affinity and accessibility) for therapeutic vascular immunotargeting to PECAM-1.

It has been reported that specific mAbs to huPECAM-1 IgD1 augments IgD1-mediated *trans-*homophilic interactions between adjacent PECAM-1 molecules [22]. Based on these observations, it stands to reason that if the binding of one mAb to PECAM-1 can increase the binding to an adjacent PECAM-1 molecule, then it may also increase binding of a second mAb directed to a different epitope, particularly in those domains that are implicated in homophilic PECAM-1 binding. Similar types of “enhanced binding” phenomena, attributed to conformational changes induced in the target molecule due to protein allostery [34]–[36], have been reported with binding of multiple ligands to isolated proteins [37], cells [38] and tissue homogenates [39]. We are observing this unusual behavior for the first time with antibodies directed to an endothelial determinant, specifically PECAM-1 which has demonstrated potential for vascular targeting of therapeutics, including immunoconjugates [19], [21], [40], fusion proteins [3], [20], [31], and nanocarriers [23].

The results presented in this report show that the binding of certain mAbs to epitopes in PECAM-1 domains 1 and 2 enhances the binding of a second paired mAb to a distinct epitope in the same domain, both *in vitro* (**Figures 4** **,** **5** **, S3, S4**) and *in vivo* (**Figure 6**). However, not all mAb pairs exhibit “collaborative enhancement” nor to the same degree. Augmentation of [^125^I]-mAb binding is most pronounced using the paired “enhancer mAb” with a higher affinity for PECAM-1 (as is the case with mAb 37 and mAb 390). This observation is likely due to the fact that lower affinity mAb have greater potential for affinity elevation, hence the more robust differences in their binding with an enhancer mAb. The innocuous effect of lower affinity mAb 62 on [^125^I]-mAb 37 binding (**Figure S3**) further suggests that a higher affinity mAb ligand drives the increase in total binding of a paired mAb to PECAM-1.

Additional studies reveal that [^125^I]-mAb affinity to PECAM-1 also increases in the presence of an enhancer mAb. This is evidenced by the 1.5−to−4−fold decrease in the apparent K_d_ when [^125^I]-mAb 62 is co-incubated with enhancer mAb 37 both in live cells and with immobilized PECAM-1 (**Table S2**). An increase in binding affinity is also implied in the left shift of the ELISA binding curve of the therapeutic 390 scFv-TM fusion construct targeted to the mAb 390 epitope of muPECAM-1 when modulated with mAb enhancer MEC13.3 (IC_50_ = 3.49 nM→0.91 nM, P<0.001) (**Figure 5A**). We hypothesized that the improved affinity combined with an enhancement in absolute 390 scFv-TM anchored to the endothelium would result in more efficient production of APC at sites of injury. Indeed, *in vitro* studies reveal a significant increase in APC generation of 390 scFv-TM paired with mAb MEC13.3 (∼6−fold, P<0.001) at much lower fusion concentrations than 390 scFv-TM alone (**Figure 5B**). The clinical and translational impact of these findings in an *in vivo* model of lung injury is of great significance and we are currently resolving this question.

Collaborative enhancement is only realized if there exists a ternary complex comprised of the mAb-ligand, the enhancer mAb-ligand, and PECAM-1; Co-IP experiments with 390 scFv-TM demonstrate that there is a complex between 390 scFv-TM/muPECAM-1/MEC13.3 mAb (**Figure 5C**). This lends further support that enhanced mAb binding and increased production of APC is mediated directly through modulation of PECAM-1 epitope engagement.

Importantly, the collaborative enhancement of muPECAM-1 immunotargeting *in vivo* was confirmed when measuring the pulmonary uptake of [^125^I]-mAb 390 and [^125^I]-mAb MEC13.3 delivered intravenously in mice (**Figure 6**). The results of *in vivo* studies in mice highlight the difficulty in predicting unambiguously the best mAb for *in vivo* immunotargeting based on *in vitro* mAb affinity and epitope accessibility from ELISA and RIA. For instance, following normalization of pulmonary uptake for residual blood levels (localization ration, LR) there is only 1.4−fold higher endothelial selectivity of [^125^I]-mAb 390 versus [^125^I]-mAb MEC13.3 (**Figure 6B**, P<0.001). This is despite mAb 390 having ∼6.4−fold higher binding affinity, albeit a 2−fold lower epitope accessibility relative to mAb MEC13.3. Co-injection of [^125^I]-mAb with PECAM-1 non-self pairs led to 2.1−fold and 1.9−fold increase in [^125^I]-mAb 390 and [^125^I]-mAb MEC13.3 lung uptake, respectively compared to [^125^I]-mAb alone (LR reaches 3.4−fold for both [^125^I]-mAbs). The innocuous effect of co-administration of muICAM-1 mAb YN1 with muPECAM-1 [^125^I]-mAbs pulmonary uptake confirms that collaborative enhancement *in vivo* is specific for anti-PECAM-1 mAb non-self pairs.

Our findings are consistent with a model in which an enhancer mAb binds to PECAM-1 to mediate collaborative enhancement of paired mAb binding *via* a single PECAM-1 molecule or through a PECAM-1-PECAM-1 homodimer. An enhancer mAb may influence intermolecular interactions between PECAM-1 molecules in the endothelial plasmalemma in many ways, including ligand-mediated disruption of homologous dimerization and oligomerization, as has been described, for example, with VEGFR [41], EGFR/HER2 receptors [42], and ACE [43], [44]. It is known that mAbs 62 and 390 can inhibit formation of homophilic PECAM-1/PECAM-1 interactions [10], [14], [22], although it is not clear if these mAbs can actually disrupt existing PECAM-1 homodimers. In theory, the binding of anti-PECAM mAbs might illicit surface exposure of additional PECAM-1 copies *via* more generalized EC activation involving cytoskeletal rearrangements [45]. The fact that EC activation by antibody-engagement of the cell adhesion molecule ICAM-1 does not enhance anti-PECAM-1 mAb binding would argue against this scenario.

The exact mechanism of antibody-mediated collaborative enhancement of PECAM-1 is worth further investigation. The fact that collaborative enhancement of mAb binding occurs *in vivo* implies that this phenomenon may be employed to further optimize vascular PECAM-1 immunotargeting of diverse therapeutic cargoes, from anti-thrombotic agents to nanocarriers carrying antioxidants.

## Materials and Methods

### Cell lines

Unless otherwise indicated, cell culture reagents were purchased from Invitrogen (Carlsbad, CA). Human umbilical vein endothelial cells (HUVECs) endogenously expressing native human PECAM-1 (huPECAM-1) were purchased from Lonza (Walkersville, MD) and maintained in EGM-2 media (Lonza) supplemented with 10% (v/v) fetal bovine serum (FBS) and 1% (v/v) penicillin (100 units/mL)/streptomycin (100 mg/mL) (P/S). Mouse pancreatic islet endothelial cells (MS1) cells endogenously expressing native mouse PECAM-1 (muPECAM-1) were obtained from the American Type Culture Collection (ATCC, Manassas, VA) and maintained in DMEM with 10% (v/v) FBS and 1% (v/v) P/S. Human malignant mesothelioma cells (REN) stably expressing recombinant mouse PECAM-1 (REN-muP) [27], were maintained in RPMI-Glutamax supplemented with 10% (v/v) FBS, 1% (v/v) P/S, and 250 µg/mL G418.

### Antibodies

Purified mAbs to huPECAM-1, mAb 62 (mouse IgG_2a_), and 37 (mouse IgG_1_), were generously provided by Dr. M. Nakada (Centocor, Malvern, PA) [22]. Rabbit anti-mouse IgG-HRP and mouse anti-rat IgG-HRP conjugates were purchased from Amersham Biosciences (Pittsburg, PA). Mouse anti-FLAG-M2-HRP mAb was purchased from Sigma-Aldrich (St-Louis, MO). The anti-mouse PECAM-1 monoclonal antibody 390 (rat IgG_2a_) [46] and MEC13.3 (rat IgG_2a_) [47] were purchased from BD Bioscience (Chicago, IL) and BioLegend (San Diego, CA), respectively. The therapeutic anti-muPECAM-1 fusion protein 390 scFv-TM (390 scFv-thrombomodulin) was produced as previously reported [31]. The control IgG Ab was an irrelevant mouse or rat IgG (Jackson Immunoresearch Laboratories, West Grove, PA).

### Radiolabeling of antiPECAM-1 mAbs

MAbs were directly radioiodinated using [^125^I]NaI (Perkin Elmer, Waltham, MA) and pre-coated Iodination tubes (Thermofisher, Waltham, MA), and purified over a 2-mL desalting column (Thermofisher). The radiolabeling efficiencies were 65–95%, and the radiochemical purity, post-purification, was >95% by the trichloroacetic acid assay. Protein concentrations were determined by NanoDrop3000 spectrophotometer (Thermofisher) and the specific activities of [^125^I]-mAb were calculated to be 5–10 µCi/µg.

### Live-cell PECAM-1-binding assays

MAbs binding to PECAM-1 on confluent live-cell monolayers was analyzed by Enzyme-Linked Immunosorbent Assay (ELISA), radioimmunoassay (RIA), and co-immunoprecipitation (co-IP) using human (HUVEC) and mouse (MS1) ECs endogenously expressing native PECAM-1, and endothelial-like human REN-muP cells [29] expressing recombinant muPECAM-1. Wild-type REN cells were used as a negative control cell-line.

#### ELISA

Cells were grown to confluence in 1% gelatin-coated 96-well plates (BD Biosciences). Monolayers were incubated with increasing concentration of mAbs in assay buffer (cell culture media with 5% FBS) at 4°C for 2 h. Cells were washed twice with assay buffer. Secondary horseradish peroxidase (HRP)-IgG antibody conjugates were added: 1∶10,000 dilution of anti-mouse-IgG (for huPECAM-1 mAbs), anti-rat-IgG (for muPECAM-1 mAbs), or anti-FLAG-M2-IgG (for 390 scFv-TM) diluted in assay buffer, followed by 1 h incubation at 4°C. Cells were washed (three times with of 3% (w/v) bovine serum albumin (BSA)/PBS) then developed with *o*-phenylenediamine (OPD; Sigma-Aldrich)/H_2_O_2_/PBS solution for 30–45 min. The reaction was quenched with the addition of 100 µL of 5 M H_2_SO_4_. Absorbance readings at 490 nm (OD_490_) were performed on a Multiskan FC Microplate reader (Thermofisher) at room temperature.

The modulation of muPECAM-1-targeted 390 scFv-TM binding in the presence of self-paired parental mAb 390 or paired mAb MEC13.3 was performed by incubating REN-muP cells with a series dilution of 390 scFv-TM co-mixed with 2−fold excess of muPECAM-1 IgG mAbs. Data were collected as described above, and the observed specific binding was plotted as a function of 390 scFv-TM added.

All ELISA binding data were analyzed using Prism 5.0 (GraphPad, San Diego, CA) software to determine relative binding affinity constants, as defined by IC_50_. Data were fit using equation (1) for the “four-parameter logisitic (4PL) non-linear regression model” most commonly used for sigmoidal curves such as ELISAs:
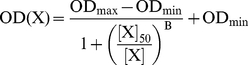
(1)OD(x) is the OD_490_ value as a function of X, the mAb concentration [mAb]. [X]_50_ is [mAb] at the inflection point of the curve when binding is half-maximal (IC_50_). B is the Hill Slope coefficient. IC_50_ values are reported as the mean ± standard deviation (SD) of three independent experiments, with each experiment performed in triplicate.

#### RIA

Cells were grown to confluence in 1% gelatin-coated 96-strip-well microplates (Corning Life Sciences, Lowell, MA). For binding assay, monolayers of cells were incubated with increasing concentration of [^125^I]-mAb (1.8 pM–5 nM in assay buffer) in quadruplicate at 4°C for 2 h. At the end of incubation, cells were washed five times with ice-cold assay buffer. The cell-associated radioactivity was measured by a gamma counter and was normalized to the total number of cells, as counted by a hemocytometer. Non-specific binding (NSB) was calculated by subtracting the total binding calculated from performing the binding assays in the presence of 100−fold excess of unlabeled protein or by subtracting radiolabeled ligand binding to wild-type cells. The data from the live-cell RIA experiments were analyzed by Scatchard analysis using Prism 5.0 (GraphPad) software to determine equilibrium binding constant and the number of functional binding sites.

The apparent binding affinity, K_d_, for specific binding was calculated using non-linear regression analysis of a one-site binding hyperbola:
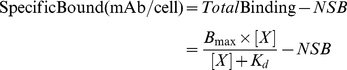
(2)where B_max_ is the maximum number of binding sites per cell at the asymptotic maximum; X is [mAb], and K_d_ is the apparent equilibrium dissociation constant. K_d_ and B_max_ values represent the mean ± SD of three or more independent experiments, and each independent experiment was performed in quadruplicate.

The modulation of [^125^I]-mAb binding in the presence of unlabeled self-paired or paired mAb was performed by incubating cells with a series dilution of nonlabeled mAb co-mixed with a fixed concentration of [^125^I]-mAb (0.3–0.6 nM) for 2 h at 4°C to allow binding. NSB was determined in the presence of 100−fold excess of unlabeled self-paired mAb. Data were collected as described above, and the observed specific binding was plotted as a function of unlabeled mAb concentration. The IC_50_ for mAb self-pairs and pairs was determined by fitting this data to a four-parameter fit (see Equation 1).

#### Co-IP

REN-muP cells were grown to confluence in a 6 well plate. Cells were incubated with MEC13.3 (50 nM), 390 scFv-TM-FLAG (25 nM), or both for 30 min at 37°C and then washed thrice to remove unbound protein. Cells were lysed at 4°C with 1 mL of RIPA buffer (Upstate, Lake Placid, NY) with protease inhibitor (Sigma-Aldrich) and then spun at 14,000×*g* for 5 min. Cell lysate supernatants were then incubated with rProtein G Agarose Beads (Invitrogen, Carlsbad, CA) overnight at 4°C to precipitate mAb MEC13.3. Beads were collected by pulse centrifugation and washed twice in ice cold RIPA buffer. Both the cell lysates and the Protein G precipitates were analyzed by SDS-PAGE and immunoblotting (IB). MuPECAM-1 was detected using polyclonal goat anti-muPECAM-1 and donkey anti-goat-HRP (both from Santa Cruz Biotech). 390 scFv-TM-FLAG was detected with anti-FLAG-M2-HRP. MAb MEC13.3 was detected with anti-rIgG-HRP. Control experiments were performed with REN wild type cells where no muPECAM-1, 390 scFv-TM, or MEC13.3 IP was detected.

### RIA with immobilized protein

Soluble recombinant (r) huPECAM-1 (extracellular domain of huPECAM-1, ∼85 kDa, Antigenix America, Huntington Station, NY), mAb 62, mAb 37, and Chrom Pure mouse IgG (mIgG, Jackson ImmunoResearch Labs, Westgrove, PA) were coated on plastic RIA 96-strip-well plates (0.32 cm^2^/well) at a concentration of 0.1 mg/mL (5 µg/well), overnight at 4°C. Wells were washed thrice with 0.05% (v/v) Tween 20 (BioRad, Hercules, CA)/PBS, and then blocked for 2 h at 4°C with 3% (w/v) BSA/PBS. Wells were incubated with increasing concentration of [^125^I]-mAb (1.8 pM–3 nM in assay buffer) in quadruplicate at 4°C for 2 h. An additional huPECAM-1 plate was treated with [^125^I]-mAb 62+50 nM mAb 37 to evaluate the changes in apparent K_d_ of [^125^I]-mAb 62 with enhancer mAb treatment. At the end of incubation, cells were washed five times with ice-cold assay buffer. Subsequent experimental work-up and data analysis follows similar methods to that described for live-cell RIA.

### Activated protein C (APC) activity assay in live-cells

Previously reported assays of APC generation on the endothelial cell surface [48], [49] were modified to allow measurement of APC generation by 390 scFv-TM bound to muPECAM-1 expressing cells. REN-muP and control REN cells were grown to confluence in 1% gelatin-coated 24-well plates (BD Biosciences). Cells were washed with serum free media then incubated with specified concentrations of 390 scFv-TM (±2−fold excess mAb MEC13.3) for 30 min at 37°C. Cells were washed three times with assay buffer (20 mM Tris, 100 mM NaCl, 1 mM CaCl_2_, 0.1% (w/v) BSA, pH 7.5) then incubated with 1 nM bovine thrombin (Sigma-Aldrich) and 100 nM protein C (Haematologic Technologies, Essex Junction, VT) in assay buffer for 1 hour at 37°C. Aliquots were removed and APC activity was measured by adding 100 nM hirudin (to inhibit thrombin; Sigma-Aldrich) and 0.5 mM of the APC substrate S-2366 (Diapharma, West Chester, OH). All samples were run in duplicate. The rate of substrate hydrolysis was measured by monitoring the change in absorbance at 405 nm over time (mOD_405_/min) at room temperature using a Multiskan FC Microplate reader. These mOD_405_/min values were subsequently converted to nmol APC using a standard curve generated using purified APC.

### Animals

Wild-type C57BL/6 female mice (16–20 g) were obtained from Jackson Laboratory (Bar Harbor, ME).

### Ethics Statement

Animals were cared for and handled in accordance with the Guide for the Care and Use of Laboratory Animals as adopted by the NIH, under a protocol approved by the University of Pennsylvania Institutional Animal Care and Use Committee (IACUC). The approved protocol number was 802060.

### In vivo targeting to the pulmonary endothelium

Mice were injected intravenously *via* jugular vein with rat muPECAM-1 [^125^I]-mAb (390 or MEC13.3) or control rat [^125^I]-IgG (*n* = 4–5 mice per group). The injected dose was constituted in 200 µL saline with 0.3% (w/v) BSA. Organs were collected at 30 min post-injection for gamma counting (Wizard Wallac 1470, Perkin Elmer). Data are expressed as % injected dose per gram of tissue (% ID/g), and are reported as the mean ± standard error of measurement (SEM) of *n* = 4–5 animals:
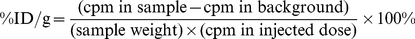
(3)


The pulmonary vasculature represents approximately 30% of total endothelial surface in the body and gets preferential perfusion by 50% of the total cardiac blood output [33], thus pulmonary uptake of the PECAM-1 targeted [^125^I]-mAb, once corrected for blood activity, is reflective of specific mAb binding to endothelial cells.

### Data analysis and statistics

All experiments were performed at least in triplicate with a minimum of three independent experiments. Results are expressed as mean ± SD unless otherwise noted. Significant differences between means were determined using one-way ANOVA followed by post-hoc Bonferroni multiple comparison test, or unpaired student *t*-test, as appropriate. P<0.05 was considered statistically significant. All curve fitting and statistical analyses was conducted using Prism 5.0 software.

## Supporting Information

Figure S1 **Schematic diagram of PECAM-1 (CD31) protein domain structure and sites of molecular binding interactions.** PECAM-1 is a 130 kDa type 1 transmembrane glycoprotein belonging to the Ig-like superfamily of cell adhesion molecules (CAM). It consists of six extracellular Ig C2-type domains defined by disulfide bonds (S-S), a short transmembrane spanning domain, and a long cytoplasmic tail containing two ITIM [51]. Ig-domains 1 and 2 are implicated in homophilic trans-binding interactions with endothelial PECAM-1 molecules on adjacent cells and with PECAM-1 on circulating leukocytes. Ig-domains 2, 3, 5, and 6 mediate heterophilic binding interactions with other cells surface antigens (e.g. CD177 on leukocytes) [16], [52]–[54].
(TIF)

Figure S2 ***In vitro*** **binding of muPECAM-1 mAbs 390 and MEC13.3 to live MS1 cells expressing endogenous muPECAM-1.** Cell surface binding of mAbs to native muPECAM-1 on live MS1 endothelial cells was determined by an ELISA-based method. Cells were incubated with shown concentrations of mAbs and incubated for 2 h at 4°C. The curves shown are from a representative experiment. The relative binding (IC_50_) of anti-PECAM-1 mAbs 390 and MEC13.3 is 0.057±0.02 nM, and 0.72±0.10 nM, respectively. The IC_50_ is reported as the mean IC_50_ value ± SD of three independent experiments performed in triplicate.
(TIF)

Figure S3 **Modulation of [^125^I]-mAb 37 binding to huPECAM-1 in live cells by self-paired and paired anti-PECAM-1 mAb co-incubation.** The modulation of PECAM-1 binding was determined after co-incubation of [^125^I]-mAb with increasing concentrations of unlabeled self-paired mAb or paired mAb for 2 h at 4°C. Binding data were plotted as [^125^I]-mAb bound per cell (mAb/cell) and data points were fit as described under “Methods.” MAb 37 competitively inhibits self-paired [^125^I]-mAb 37 binding to HUVEC. At variance, mAb 62 does not affect paired [^125^I]-mAb 37 binding.
(TIF)

Figure S4 **[^125^I]-mAbs 62 and 37 bind to immobilized rhuPECAM-1, but have no cross-reactivity with mAb 62, mAb 37, and control mIgG.** The binding of [^125^I]-mAbs 62 and 37 to immobilized self-paired and paired mAb were performed as described under “Methods.” RIA wells coated with rhuPECAM-1 and mIgG served as positive and negative controls for [^125^I]-mAb 62 (**A**) and [^125^I]-mAb 37 (**A**) binding, respectively. (**C**) Binding data was re-plotted as [^125^I]-mAb bound as % of input at maximal input dose. [^125^I]-MAbs have no difference in non-specific binding to mAb-coated wells, whereas binding to rhuPECAM-1 coated well is significantly higher than all IgG-coated wells (***, P<0.001).
(TIF)

Figure S5 **Modulation of [^125^I]-mAb 390 and MEC13.3 binding to endogenous muPECAM-1 in live MS1 cells.** Competitive inhibition curves were obtained with self-paired [^125^I]-mAb 390/mAb 390, and [^125^I]-mAb MEC13.3/mAb MEC13.3 mixes. Collaborative binding enhancement was observed for both mAb pairs, i.e., [^125^I]-mAb 390/mAb MEC13.3 and [^125^I]-mAb MEC13.3/mAb 390, with approximately 1.3−fold and 3−fold binding enhancement over solo binding, respectively.
(TIF)

Table S1 **Binding parameters of anti-PECAM-1 [^125^I]-mAbs 390 and MEC13.3 to live cells expressing mouse PECAM-1.** Binding affinity (K_d_) and number of binding sites (B_max_) of [^125^I]-mAb to REN-mPECAM-1 cells or MS1 cells. Note that total binding was corrected for NSB using REN cells (for REN-muP cells) or with 100−fold excess unlabeled mAb (for MS1 cells). Results were determined by three independent RIA experiments performed in quadruplicate, with data expressed as mean ± S.D.
(TIF)

Table S2 **Modulation of binding affinity of anti-huPECAM-1 [^125^I]-mAbs 62 following co-incubation with enhancer mAb 37.** Binding affinity (K_d_) of [^125^I]-mAb 62 to huPECAM-1 on live HUVECs or to immobilized rhuPECAM-1 is studied alone or in the presence of 50 nM mAb 37. Note that total binding was corrected for NSB using 100−fold excess unlabeled mAb 62. Co-treatment of HUVECs with [^125^I]-mAb 62 and mAb 37 led to a 1.4−fold increase in binding affinity over solo binding, whereas the binding affinity increases nearly four−fold following collaborative enhancement with immobilized rhuPECAM-1. Results were determined by three independent RIA experiments performed in quadruplicate, with data expressed as mean ± S.D.
(TIF)
