# Supplementary figures and images for: Collaborative Enhancement of Antibody Binding to Distinct PECAM-1 Epitopes Modulates Endothelial Targeting
Source: PLoS One. 2012 Apr 13;7(4):e34958. doi: 10.1371/journal.pone.0034958 (PMC3325922; doi:10.1371/journal.pone.0034958)

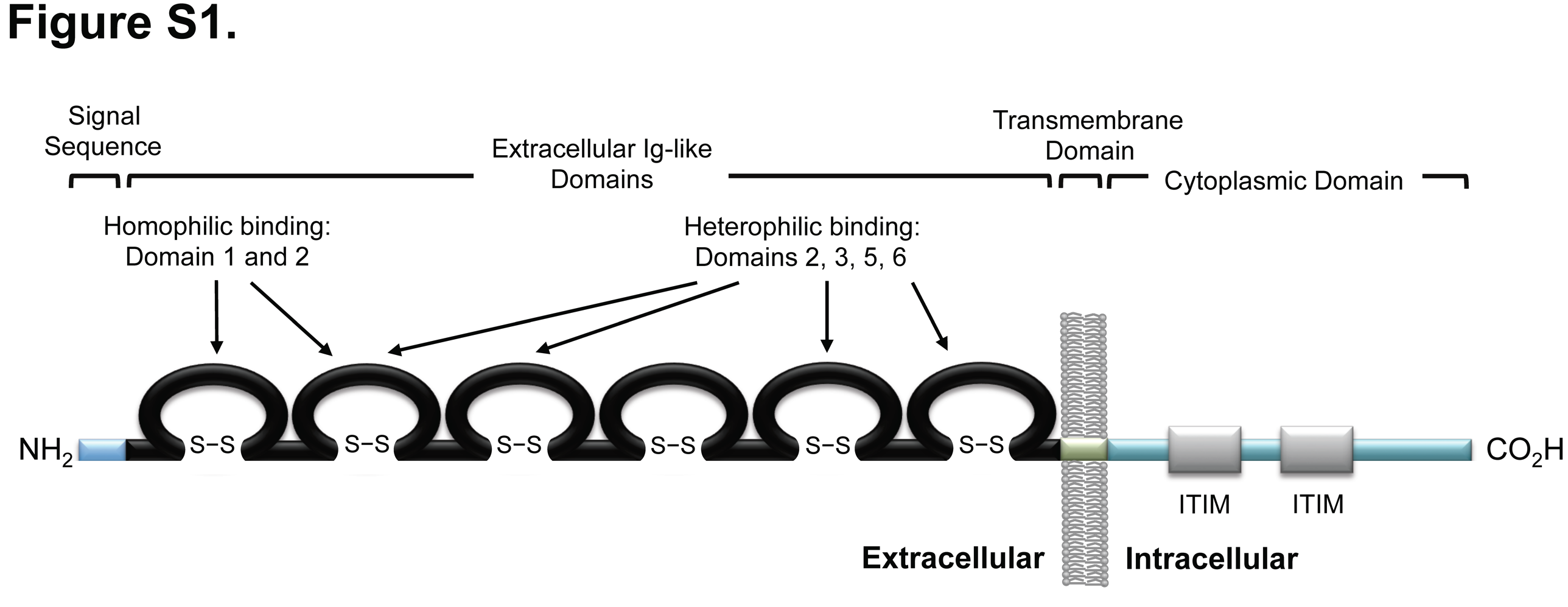

Supplement: Figure S1 — Schematic diagram of PECAM-1 (CD31) protein domain structure and sites of molecular binding interactions. PECAM-1 is a 130 kDa type 1 transmembrane glycoprotein belonging to the Ig-like superfamily of cell adhesion molecules (CAM). It consists of six extracellular Ig C2-type domains defined by disulfide bonds (S-S), a short transmembrane spanning domain, and a long cytoplasmic tail containing two ITIM [51]. Ig-domains 1 and 2 are implicated in homophilic trans-binding interactions with endothelial PECAM-1 molecules on adjacent cells and with PECAM-1 on circulating leukocytes. Ig-domains 2, 3, 5, and 6 mediate heterophilic binding interactions with other cells surface antigens (e.g. CD177 on leukocytes) [16], [52]–[54]. (TIF) [file pone.0034958.s001.tif]

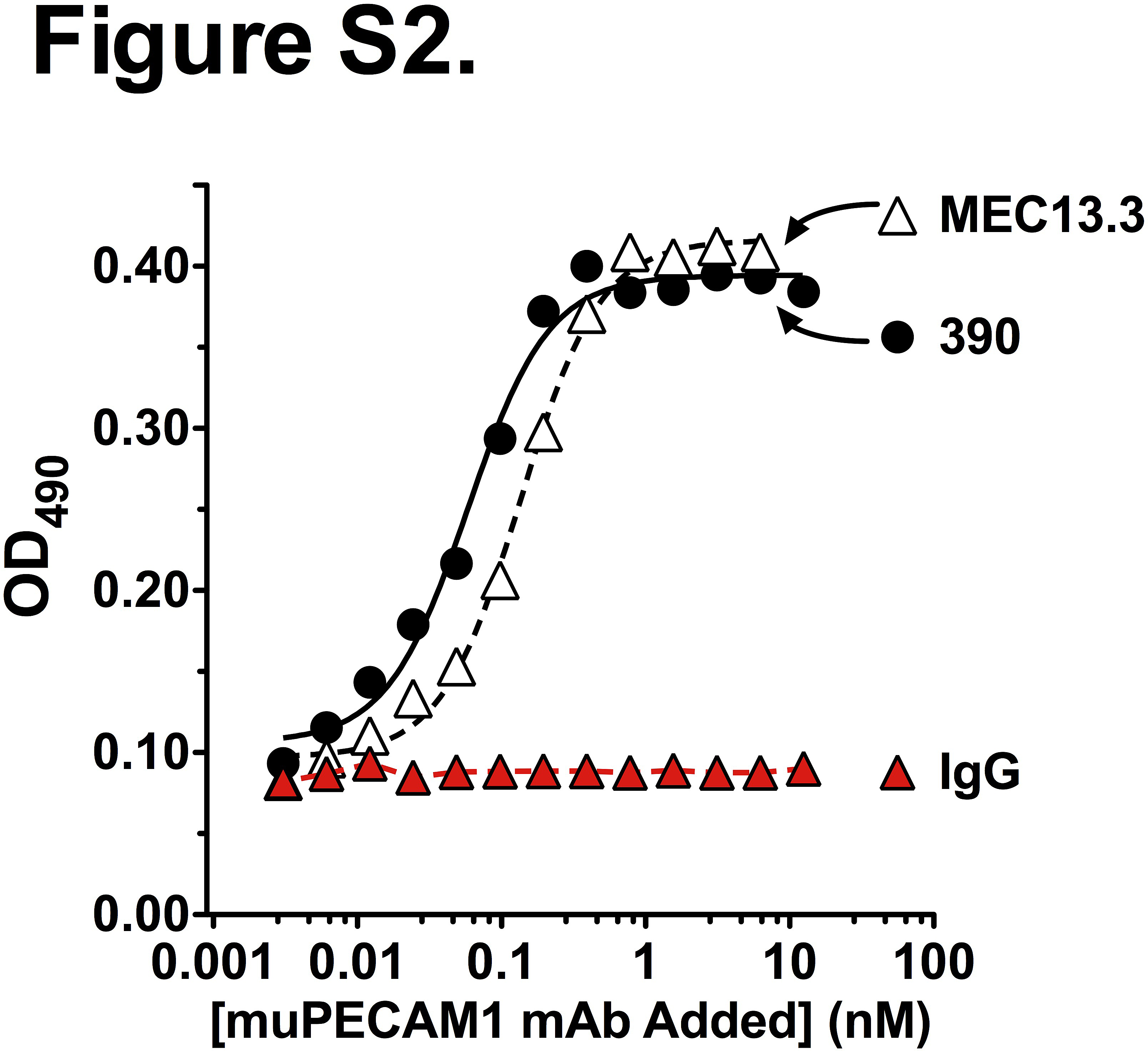

Supplement: Figure S2 — In vitro binding of muPECAM-1 mAbs 390 and MEC13.3 to live MS1 cells expressing endogenous muPECAM-1. Cell surface binding of mAbs to native muPECAM-1 on live MS1 endothelial cells was determined by an ELISA-based method. Cells were incubated with shown concentrations of mAbs and incubated for 2 h at 4°C. The curves shown are from a representative experiment. The relative binding (IC50) of anti-PECAM-1 mAbs 390 and MEC13.3 is 0.057±0.02 nM, and 0.72±0.10 nM, respectively. The IC50 is reported as the mean IC50 value ± SD of three independent experiments performed in triplicate. (TIF) [file pone.0034958.s002.tif]

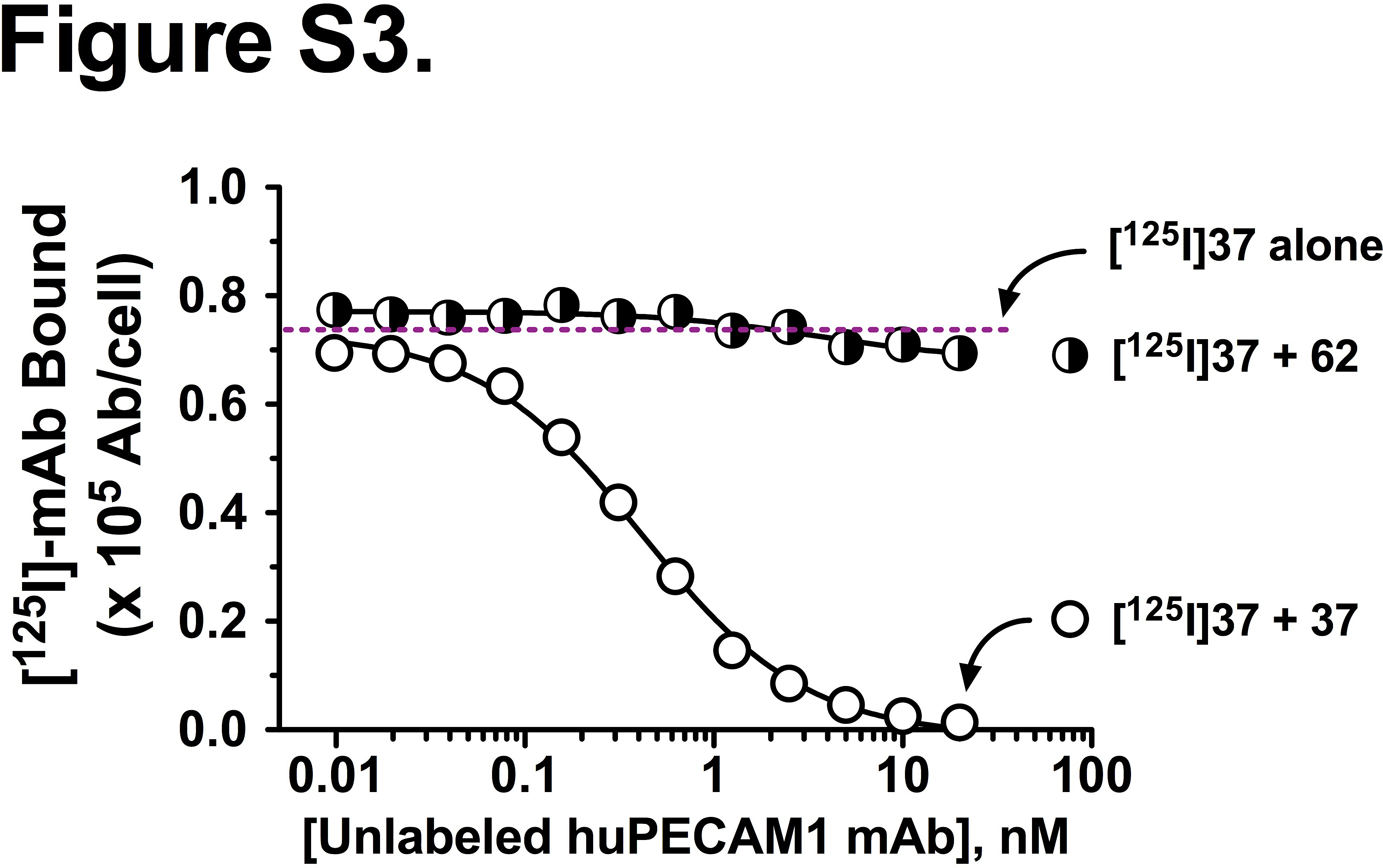

Supplement: Figure S3 — Modulation of [125I]-mAb 37 binding to huPECAM-1 in live cells by self-paired and paired anti-PECAM-1 mAb co-incubation. The modulation of PECAM-1 binding was determined after co-incubation of [125I]-mAb with increasing concentrations of unlabeled self-paired mAb or paired mAb for 2 h at 4°C. Binding data were plotted as [125I]-mAb bound per cell (mAb/cell) and data points were fit as described under “Methods.” MAb 37 competitively inhibits self-paired [125I]-mAb 37 binding to HUVEC. At variance, mAb 62 does not affect paired [125I]-mAb 37 binding. (TIF) [file pone.0034958.s003.tif]

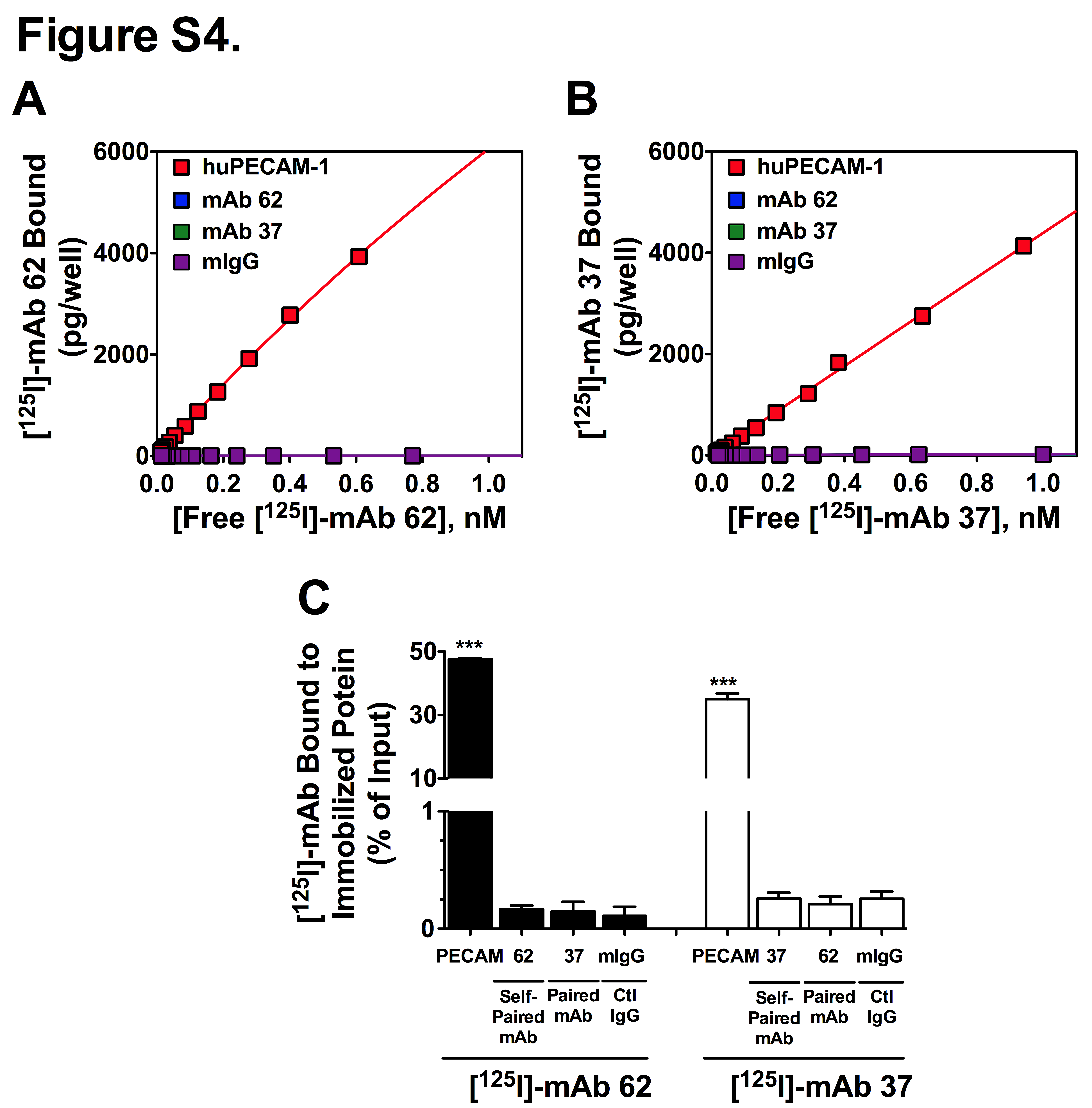

Supplement: Figure S4 — [125I]-mAbs 62 and 37 bind to immobilized rhuPECAM-1, but have no cross-reactivity with mAb 62, mAb 37, and control mIgG. The binding of [125I]-mAbs 62 and 37 to immobilized self-paired and paired mAb were performed as described under “Methods.” RIA wells coated with rhuPECAM-1 and mIgG served as positive and negative controls for [125I]-mAb 62 (A) and [125I]-mAb 37 (A) binding, respectively. (C) Binding data was re-plotted as [125I]-mAb bound as % of input at maximal input dose. [125I]-MAbs have no difference in non-specific binding to mAb-coated wells, whereas binding to rhuPECAM-1 coated well is significantly higher than all IgG-coated wells (***, P<0.001). (TIF) [file pone.0034958.s004.tif]

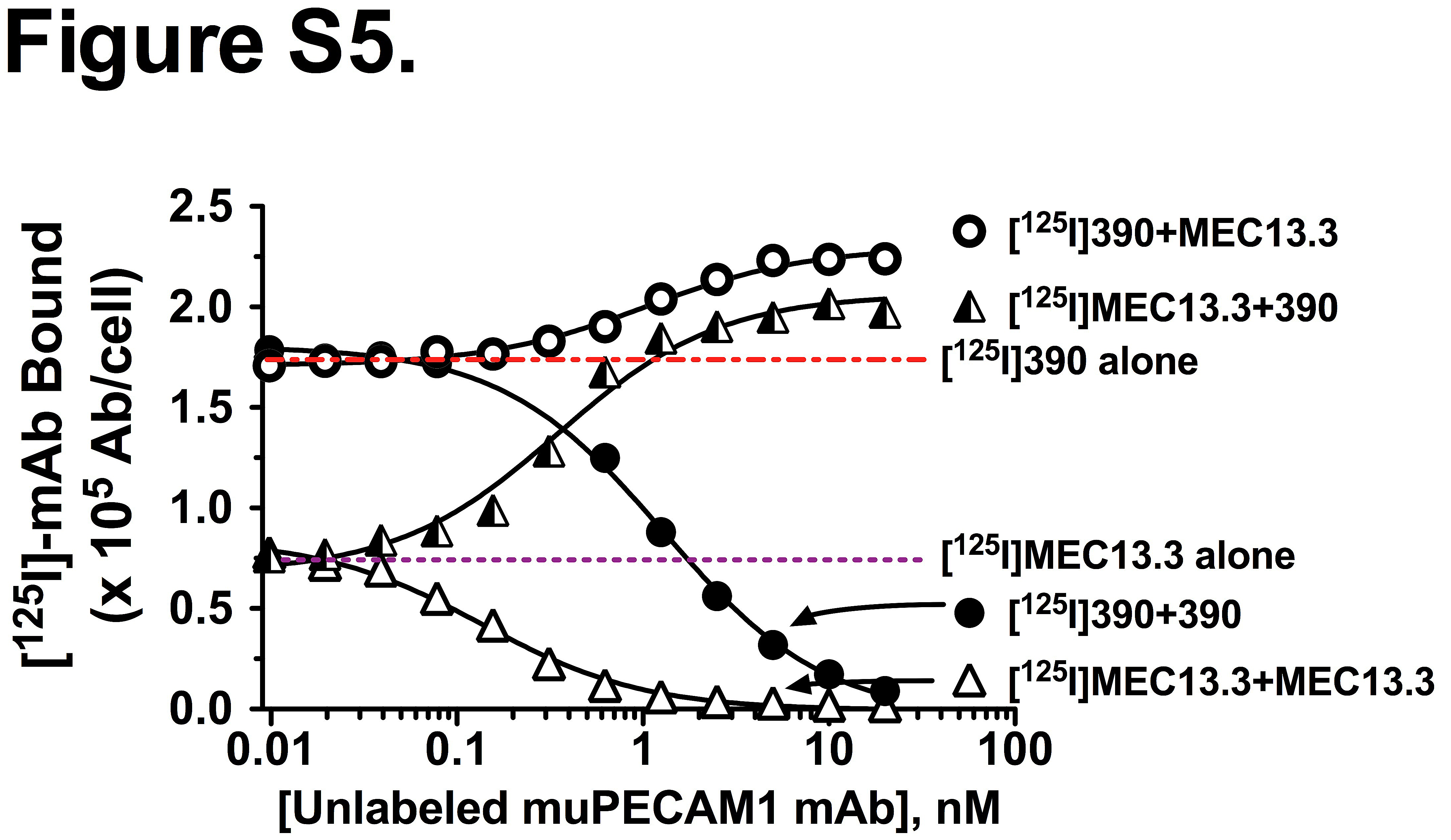

Supplement: Figure S5 — Modulation of [125I]-mAb 390 and MEC13.3 binding to endogenous muPECAM-1 in live MS1 cells. Competitive inhibition curves were obtained with self-paired [125I]-mAb 390/mAb 390, and [125I]-mAb MEC13.3/mAb MEC13.3 mixes. Collaborative binding enhancement was observed for both mAb pairs, i.e., [125I]-mAb 390/mAb MEC13.3 and [125I]-mAb MEC13.3/mAb 390, with approximately 1.3−fold and 3−fold binding enhancement over solo binding, respectively. (TIF) [file pone.0034958.s005.tif]

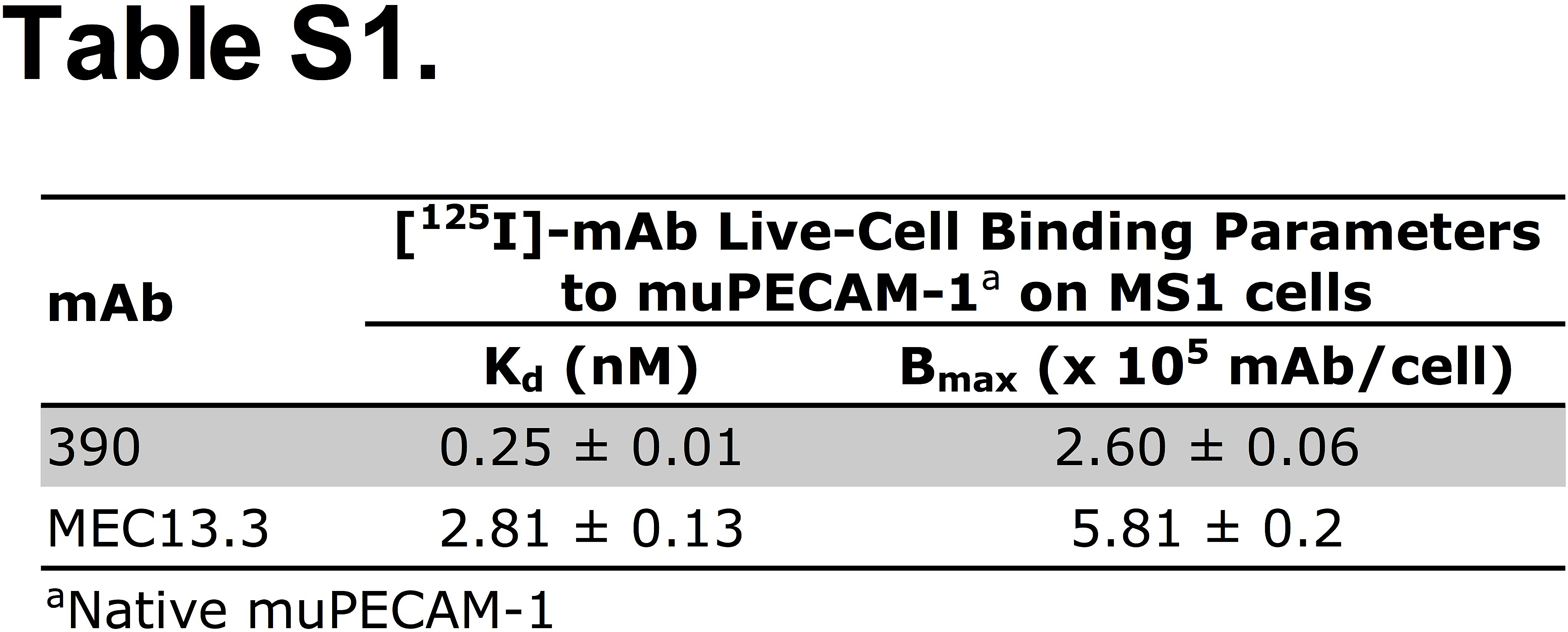

Supplement: Table S1 — Binding parameters of anti-PECAM-1 [125I]-mAbs 390 and MEC13.3 to live cells expressing mouse PECAM-1. Binding affinity (Kd) and number of binding sites (Bmax) of [125I]-mAb to REN-mPECAM-1 cells or MS1 cells. Note that total binding was corrected for NSB using REN cells (for REN-muP cells) or with 100−fold excess unlabeled mAb (for MS1 cells). Results were determined by three independent RIA experiments performed in quadruplicate, with data expressed as mean ± S.D. (TIF) [file pone.0034958.s006.tif]

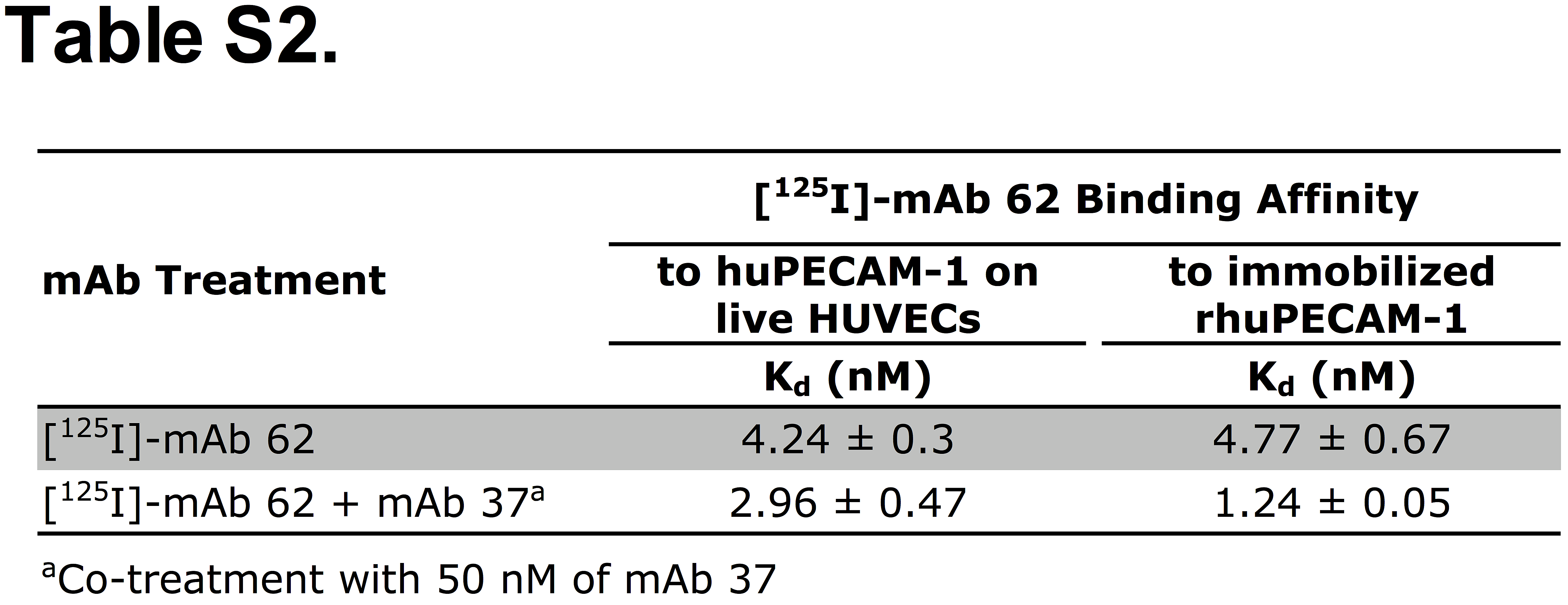

Supplement: Table S2 — Modulation of binding affinity of anti-huPECAM-1 [125I]-mAbs 62 following co-incubation with enhancer mAb 37. Binding affinity (Kd) of [125I]-mAb 62 to huPECAM-1 on live HUVECs or to immobilized rhuPECAM-1 is studied alone or in the presence of 50 nM mAb 37. Note that total binding was corrected for NSB using 100−fold excess unlabeled mAb 62. Co-treatment of HUVECs with [125I]-mAb 62 and mAb 37 led to a 1.4−fold increase in binding affinity over solo binding, whereas the binding affinity increases nearly four−fold following collaborative enhancement with immobilized rhuPECAM-1. Results were determined by three independent RIA experiments performed in quadruplicate, with data expressed as mean ± S.D. (TIF) [file pone.0034958.s007.tif]
